# Supplementary figures and images for: Recombinant Rift Valley fever viruses encoding bluetongue virus (BTV) antigens: Immunity and efficacy studies upon a BTV-4 challenge
Source: PLoS Negl Trop Dis. 2020 Dec 4;14(12):e0008942. doi: 10.1371/journal.pntd.0008942 (PMC7744063; doi:10.1371/journal.pntd.0008942)

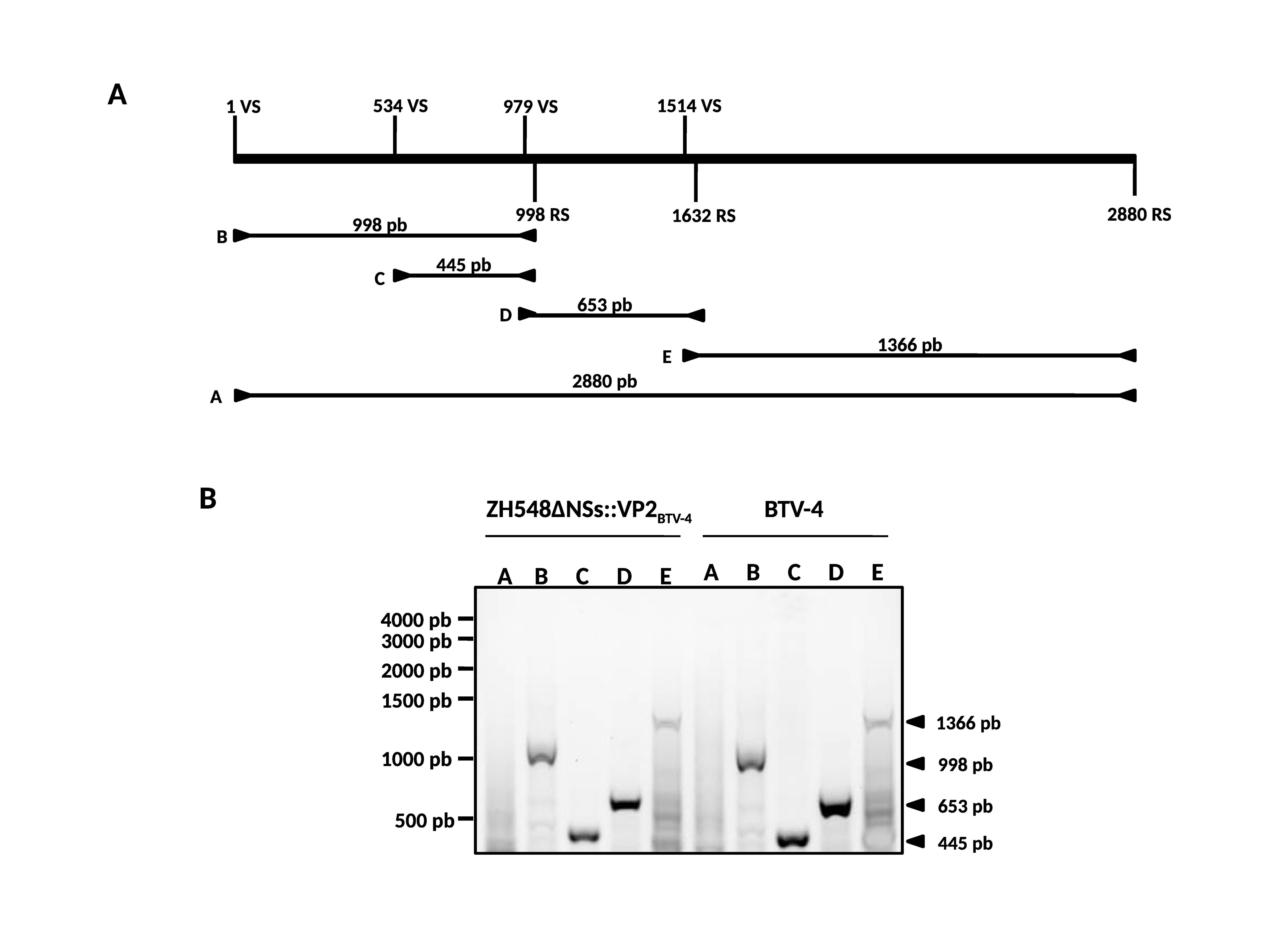

Supplement: S1 Fig — A. Schematic of the amplification strategy. Different size VP2 fragments were amplified from cDNA templates. cDNA primers used for first strand synthesis are depicted in red. B. Detection of specific fragments by agarose gel electrophoresis. (TIF) [file pntd.0008942.s001.tif]

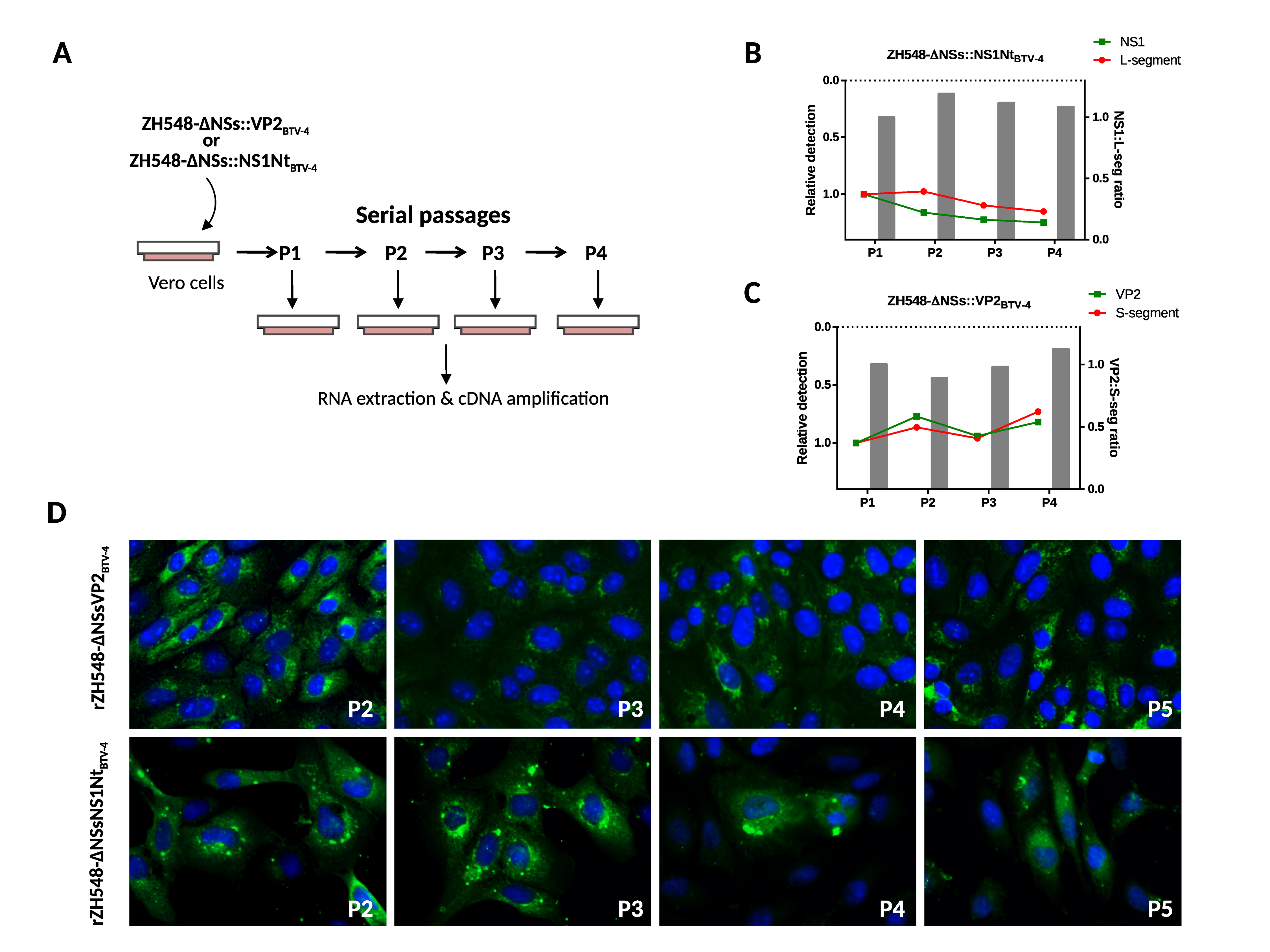

Supplement: S2 Fig — A. Schematic of the passage history of the recombinant viruses. Vero cell monolayers were infected with of a MOI of 1 with each virus from a first (P1), second (P2), third (P3), or fourth (P4) passage. B. Relative detection of L segment RNA (red line) and NS1Nt gene (green) from ZH548-ΔNSs::NS1NtBTV-4 by RT-qPCR (left Y axis). Bars represent the L/NS1 ratio from Ct values (right Y axis). C. Relative detection of RVFV nucleoprotein N (red) and BTV VP2 (green) genes from ZH548-ΔNSs::VP2BTV-4 by PCR. Bars represent the S-segment/VP2 ratio from densitometry values of gel-based DNA fragments of 521bp and 653bp corresponding to the N and VP2 genes respectively. D. Detection of VP2 and NS1 Nt transgenes by immunofluorescence of Vero cells infected with serially passaged recombinant virus (p2 to p5). Mouse anti-VP2 polyclonal serum was used for VP2 detection and an anti-V5 mAb was used for the detection of NS1Nt (green fluorescence). Nuclei were visualized by DAPI staining. (TIF) [file pntd.0008942.s002.tif]

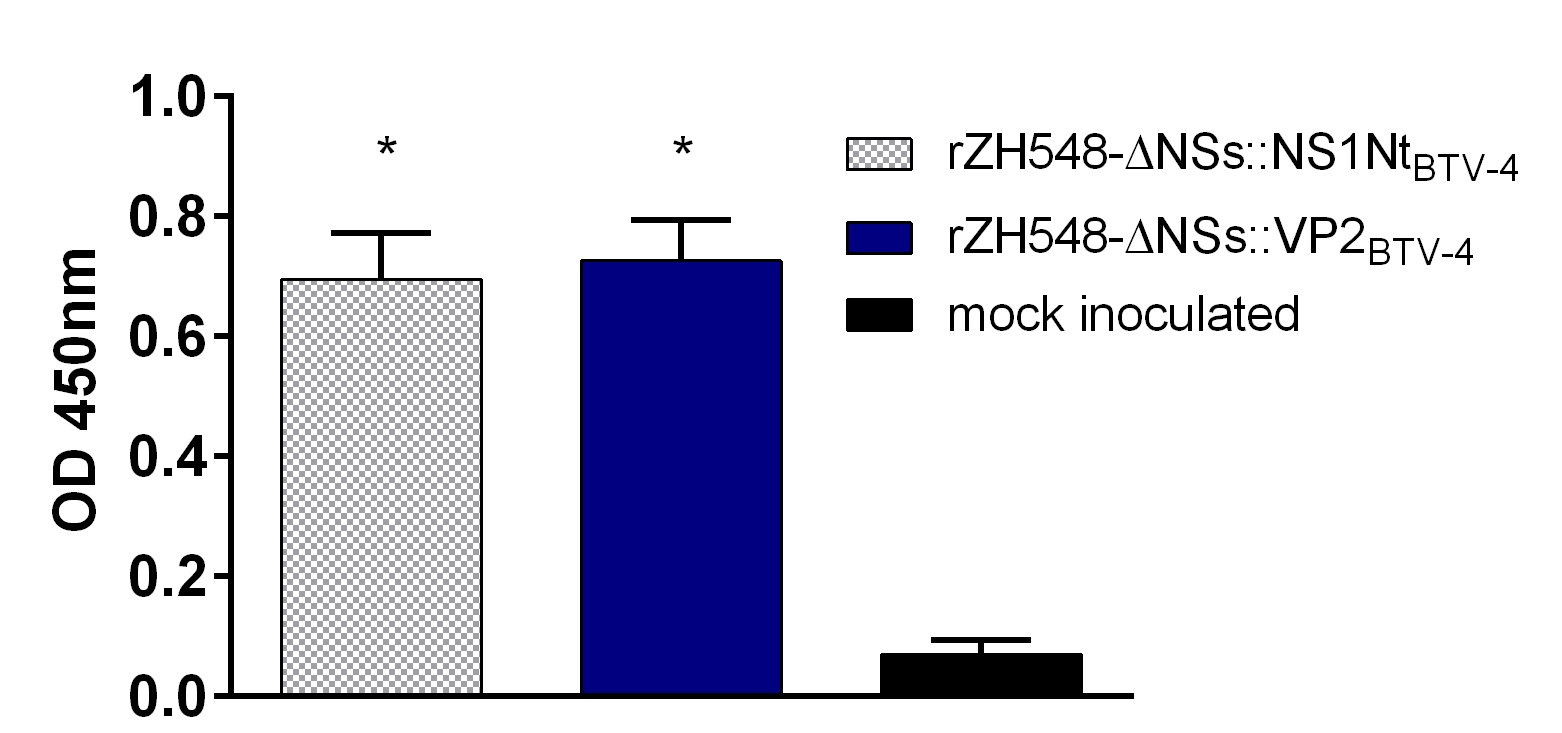

Supplement: S3 Fig — Detection of anti-RVFV nucleoprotein N antibodies 14 days upon inoculation with 107 pfu of rZH548-ΔNSs::VP2BTV-4 and rZH548-ΔNSs::NS1NtBTV4 or PBS (mock inoculated control) by ELISA. Bars represent median plus interquartile range. *P<0.05 (Anova, non-parametric Kruskall-Wallis test). (TIF) [file pntd.0008942.s003.tif]

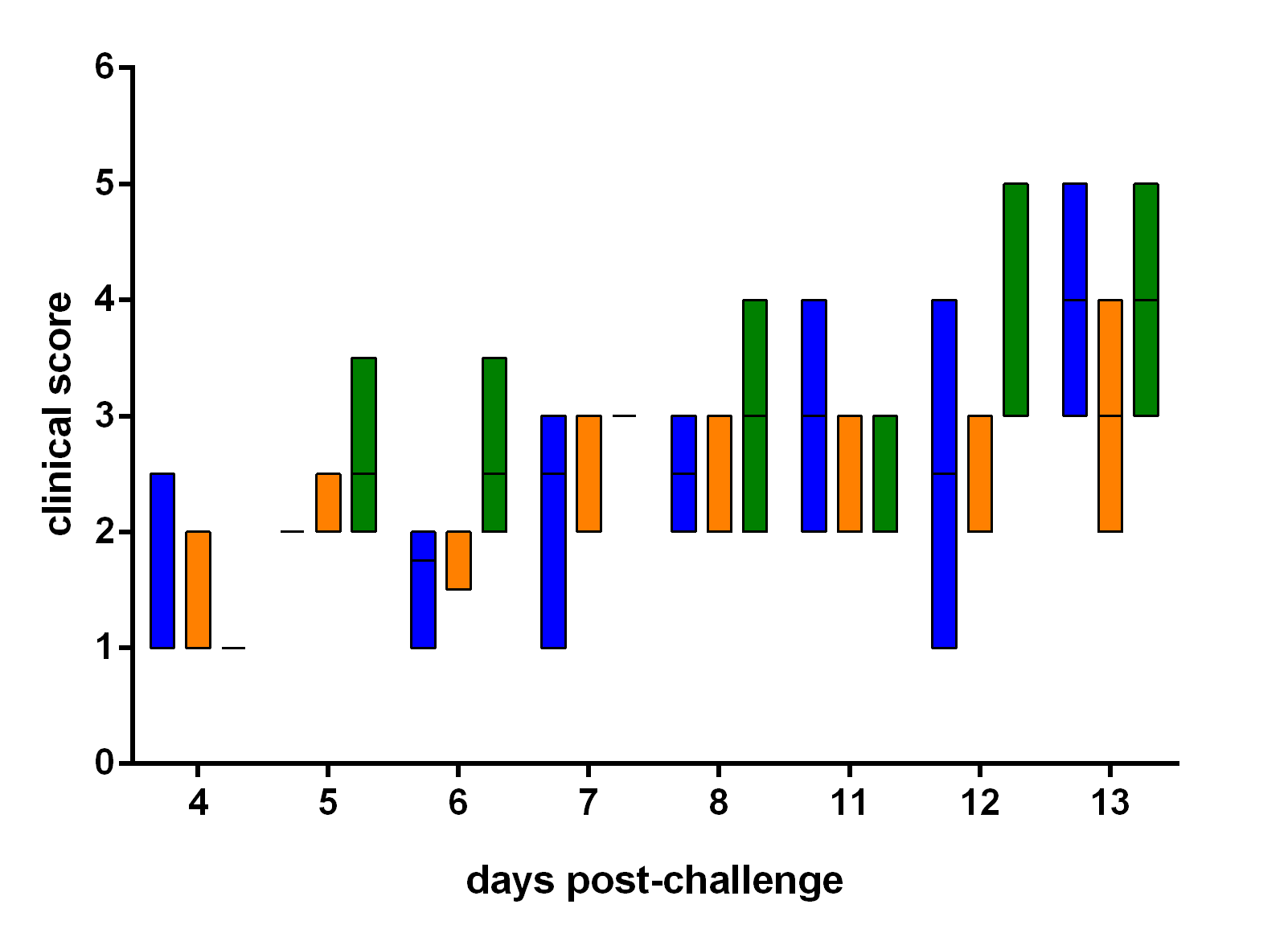

Supplement: S4 Fig — Clinical display of vaccinated sheep groups at different days after challenge. Scoring was based on the severity of signs observed by blinded veterinary personnel (see Methods). The graph represent the mean (horizontal lines) and min to max values (bars). (TIF) [file pntd.0008942.s004.tif]

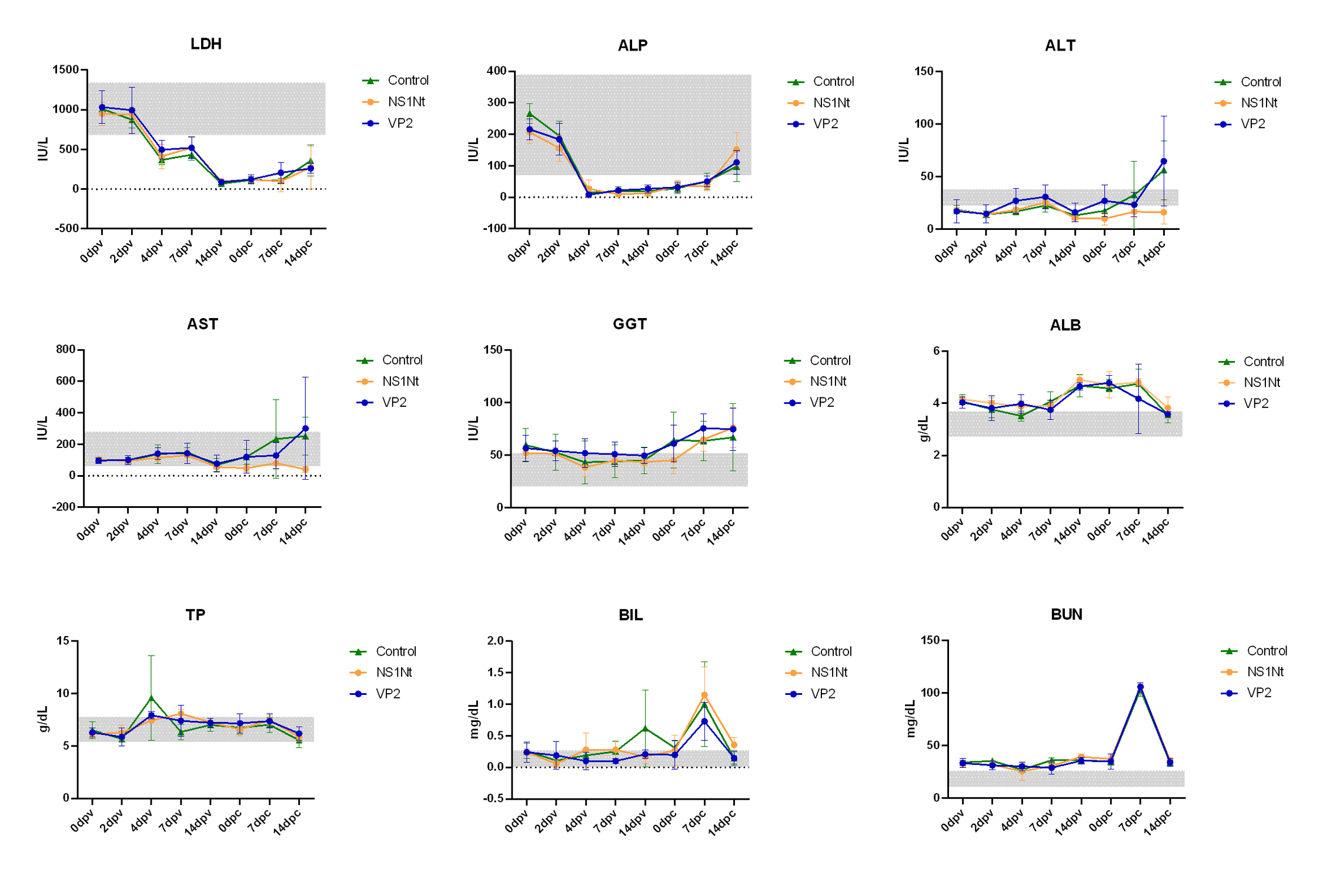

Supplement: S5 Fig — The following biochemical parameters were measured in serum taken at the indicated days post vaccination (dpv) or post challenge (dpc): AST: Aspartate aminotransferase. ALT: Alanine aminotransferase. LDH: Lactate dehydrogenase. GGT: Gamma-glutamyltransferase. ALP: Alkaline phosphatase. ALB: serum albumin. BIL: serum bilirubin. BUN: serum urea nitrogen. TP: serum total protein. Error bars represent SD. Shadowed areas denote normal sheep enzyme values as described previously [59]. (TIF) [file pntd.0008942.s005.tif]
