# Supplementary material for: Recombinant Rift Valley fever viruses encoding bluetongue virus (BTV) antigens: Immunity and efficacy studies upon a BTV-4 challenge
Source: PLoS Negl Trop Dis. 2020 Dec 4;14(12):e0008942. doi: 10.1371/journal.pntd.0008942 (PMC7744063; doi:10.1371/journal.pntd.0008942)
Supplement: S1 Table — (DOCX) [file pntd.0008942.s006.docx]

Supporting information

**TABLE 1**

List of primers used for plasmid construction and RT-PCR analysis

| **Primer** | **Sequence (5’-3’)** |
| --- | --- |
| **VP2_NcoI_fwd** | catgCCATGGatggaggagtttgtcattcc |
| **VP2_XhoI_rev** | ccgCTCGAGctaaacgttgagtaatttcg |
| **NS1_NcoI_fwd** | catgCCATGGatggagcgctttttgagaaa |
| **NS1_XhoI_rev** | ccgCTCGAGctaatattccatccacatctga |
| **NS1Nt_XhoI_rev** | ccgCTCGAGctaacctgttggaacccttc |
| **NS1_fw_GxC** | gcggtgcggggtgcaactgctggaggattaccg |
| **NS1_rev_GxC** | cggtaatcctggagcagttgcaccccgcaccgc |
| **NS1Nt_V5tag_fwd** | ctactaggcctagattagatgtggatggaatattag |
| **NS1Nt_V5tag_rev** | ggggttgggtatacctgttggaaccct |
| **L-seg fwd** | ttctttgcttctgataccctctgt |
| **L-seg rev** | gttccacttccttgcatcatctg |
| **L-seg Taqman probe** | 6-FAM-ttgcacaagtccacacaggcccct-BHQ1 |
| **Ndelta9 fwd** | tcgaaagaaggcaaa |
| **ss1** | agccacttaggctgctgtcttgt |
| **VP2 fwd 979** | cggacgagtggggctgccac |
| **VP2 rev 1632** | cctattccacggatcggccg |
| **BTS5F1** | ggcaacyaccaaacatgga |
| **BTS5R76-57** | aaagtyctcgtggcattwgc |
| **BTV4-1 VS** | cgcccgggatggaggagtttgtcattcc |
| **BTV4-534 VS** | ccacgtacttcaaggcgctgc |
| **BTV4-979 VS** | cggacgagtggggctgccac |
| **BTV4-1514 VS** | cggaactagtgttcccaaac |
| **BTV4-998 RS** | gtggcagccccactcgtccg |
| **BTV4-1632 RS** | cctattccacggatcggccg |
| **BTV4-2852 RS** | cgcccgggctaaacgttgagtaatttcg |
|  |  |
