# Supplementary material for: Recombinant Rift Valley fever viruses encoding bluetongue virus (BTV) antigens: Immunity and efficacy studies upon a BTV-4 challenge
Source: PLoS Negl Trop Dis. 2020 Dec 4;14(12):e0008942. doi: 10.1371/journal.pntd.0008942 (PMC7744063; doi:10.1371/journal.pntd.0008942)
Supplement: Attachment — Submitted filename: Response letter plosntd.docx [file pntd.0008942.s008.docx]

**When you are ready to resubmit, please upload the following:**

**[1] A letter containing a detailed list of your responses to the review comments and a description of the changes you have made in the manuscript. Please note while forming your response, if your article is accepted, you may have the opportunity to make the peer review history publicly available. The record will include editor decision letters (with reviews) and your responses to reviewer comments. If eligible, we will contact you to opt in or out.**

**[2] Two versions of the revised manuscript: one with either highlights or tracked changes denoting where the text has been changed; the other a clean version (uploaded as the manuscript file).**

**Important additional instructions are given below your reviewer comments.**

**Please prepare and submit your revised manuscript within 60 days. If you anticipate any delay, please let us know the expected resubmission date by replying to this email. Please note that revised manuscripts received after the 60-day due date may require evaluation and peer review similar to newly submitted manuscripts.**

**Thank you again for your submission. We hope that our editorial process has been constructive so far, and we welcome your feedback at any time. Please don't hesitate to contact us if you have any questions or comments.**

**Response letter to Manuscript ID: PNTD-D-20-00574.**

We want to thank beforehand all the reviewers’ for their comments and constructive suggestions. We have used their comments (in blue) as a template to answer (in red) the specific requirements and comments.

**Methods**


-Are the objectives of the study clearly articulated with a clear testable hypothesis stated?

-Is the study design appropriate to address the stated objectives?

-Is the population clearly described and appropriate for the hypothesis being tested?

-Is the sample size sufficient to ensure adequate power to address the hypothesis being tested?

-Were correct statistical analysis used to support conclusions?

-Are there concerns about ethical or regulatory requirements being met?


Reviewer #1: yes

Reviewer #2: 1. Figures 4C, 6B, and 9B: Authors showed “log total pfu” along with Ct value of qRT-PCR. It is impossible to convert RNA copy number (a mixture of genomic RNA and mRNA) into plaque forming unit, which is the infectious virus unit. Please correct them into “Viral RNA copies” based on the comparison with standard curve using control viral RNA. The calculation should also be described in the materials and method. The graphs plotted Ct (Cq) values. For reference purposes we intended to show the approximate equivalence (in plaque forming units) to detected Cts. These equivalences were calculated by an independent assay, made by spiking known amounts of virus (in pfus) from a titrated virus inoculum in naïve mouse blood, using identical blood volumes as those used to perform the RNA extraction procedure for the RT-qPCR assay. We realized this methodology was not fully detailed in the manuscript and therefore it may have induced to confusion. We have included this information in the Methods section. Also, the legend of the right Y-axis has been changed to “log_10_ pfu equivalents”.
2. Figures 5B, 8A, 8B should clarify the base of log. We have corrected all figures to show that the base of the log is 10 (log_10_)
3. Figure 9: The comparison of two values in the experiment with three or more groups should be made by ANOVA. We have performed, as suggested, the ANOVA comparisons for the three groups. This has been detailed in figure 9 legend.
Reviewer #3: This paper describes the generation of a recombinant RVFV vaccine vector also designed to serve as a vaccine against BTV. The authors generated recombinant RVFV vaccine candidates encoding BTV4 VP2 or a truncated form of BTV4 NS1 called NS1Nt using reverse genetics. Both rescued viruses were shown to express their respective genes by immunofluorescence of infected cultures using specific antibodies. NS1Nt could be detected in infected cell lysates whereas degraded VP2 proteins were detected using immunoprecipitation. Growth kinetics and end titres as well as plaque morphologies were compared with the control. Stability of the viruses while passaging through cells was confirmed.
Immunisation of Balb/C mice with the rescued viruses did not affect the antibody response against RVFV N. The VP2 immunisation showed a dose-dependent response on anti-N antibody production but a challenge with a lethal dose of RVFV showed survival of all mice given different doses and corresponding levels of viraemia with dose. Analysis of the VP2 antibody response in the same mice showed a dose-dependent VP2 antibody-specific response increasing over time and after a BTV4 boost. Neutralising antibodies also showed a dose-dependent response with a decrease after 21 days and a significant increase after the BTV4 boost. Only passive immunisation of IFNAR mice with serum from mice vaccinated with the VP2 vaccine and BTV4 virus boost resulted in their survival with correspondingly low viraemia levels. ICCS and ELISPOT results from serum of mice immunised with NS1Nt and a boost with BTV4 showed significant levels of cellular stimulation with lower levels from those receiving the NS1Nt only immunisation.
Sheep immunised with either the VP2 or NS1Nt vaccines showed significant levels of neutralising antibodies for VP2 and none for NS1Nt as well as high levels for RVFV neutralising antibodies for both vaccines. After a challenge with BTV8, lower levels of pyrexia, viraemia and anti-VP7 antibodies were observed for both VP2 and NS1Nt-vaccinated sheep.

The methods are extremely well written bar a few grammatical errors and it would be very easy to repeat these experiments - minor revision. We thank very much this appreciation. We have tried to correct grammatical errors as much as possible

--------------------

**Results**


-Does the analysis presented match the analysis plan?

-Are the results clearly and completely presented?

-Are the figures (Tables, Images) of sufficient quality for clarity?


Reviewer #1: yes

Reviewer #2: 1. In sheep, the protective efficacy of two candidate vaccines against BTV was not clear, other than marginal reduction of viral replication judged from the RNA level. Apparently, this limited protective efficacy against BTV VP2 is due to the truncation of VP2 gene. Figure 2C showed that BTV VP2 proteins was largely truncated, although authors speculated that VP2 proteins were rapidly processed by specific proteases (line 602). The RVFV S-segment NSs gene cassette apparently has a size limitation to retain foreign gene. To demonstrate that majority of viral population retains the full-length of VP2 gene in the S-segment, Northern blot of chimeric S-segment RNA is essential. It is also important to characterize the sequence of remaining VP2 gene, to explain the 25kD size VP2. We agree with the reviewer in that the efficacy in sheep after a single dose was not fully satisfactory requiring further optimization. We understand that this reviewer suspects that our RVFV-VP2 recombinant virus encodes a truncated VP2 sequence, giving rise to a 25kDa protein. Unfortunately, time constraints impeded us to have ready the Northern blot technique as suggested, since we need to adapt classical ^32^P isotopic probe labelling protocols to non-radioactive probe generation. Instead, we performed a quicker alternative to test the integrity of the VP2 gene transcripts. We thus performed PCR amplification of overlapping fragments covering the full VP2 gene sequence in both RVFV-VP2 and BTV infected cells using extracted RNA. The data shows an identical pattern profile and these data has been included in a new supplemental figure. In addition, the overlapping fragments were also Sanger sequenced, to rule out the presence of frame shifts, confirming the presence of a continuous ORF. Nonetheless, we are pursuing to set up the conditions for performing Northern blot technique, since we understand this technique would be also very informative. We rather believe that upon expression in RVFV infected cells, VP2 is largely unstable giving rise to the detection of several degradation bands detected by the immunoprecipitation assay. This was in agreement with the pattern of VP2 expression observed in cells infected with other heterologous viral expression platforms, such as MVA, as stated in the text. On the other hand, the neutralization data support the expression of native VP2 epitopes since the induction of neutralizing antibodies against BTV relies in the trimeric structure of VP2 proteins. If only partially truncated VP2 protein was expressed, it would be less likely to detect the induction of neutralizing antibodies. The suggestion to investigate the nature of the 25kDa is very good and we have plans to have this properly addressed by collaborating with a proteomics lab. But, again this will need more time that exceed the requirements for this stage of publication.
2. AST and ALT values in Figure S3B may be included in the result, if partial efficacy of candidate vaccine is discussed. This observation has been included in the Results (lines 552-555). The lower levels of both hepatic enzymes in sheep vaccinated with the NS1Nt constructs could be indicative of less cellular damage.
Reviewer #3: The analysis mostly matches the plan. There are some results that are not discussed - see Summary and General Comments
Results are very clearly presented.
Images are not very good quality - it is hard to discern the differences between the plaque morphologies of figure 3b and fig 2a is also not very clear. The quality of the plaque images has been improved by performing new plaque assays and by increasing the image resolution.

--------------------

**Conclusions**


-Are the conclusions supported by the data presented?

-Are the limitations of analysis clearly described?

-Do the authors discuss how these data can be helpful to advance our understanding of the topic under study?

-Is public health relevance addressed?


Reviewer #1: No.
Major comments:
• The authors have used the incorrect term “chimeric” in the title and throughout the manuscript. The definition of chimera is a combination of two or more different species of which the parts are essential for the chimeric organism, here a chimeric virus. The described RVFV expressing GFP or a gene originating from BTV is not depending on NS1t or VP2 of BTV, and thus not a chimeric virus. Instead, the described viruses are viral vector vaccine candidates, in more detail duo of bivalent vaccine candidates potentially protecting animals against both RVF and BT. The term ”chimeric” should be removed throughout the manuscript and replaced by a an appropriate term, like bivalent vaccine or duo vaccine. We indeed had several reasonable doubts about the proper use of the word “chimeric”. We thank the reviewer for the clarification to which we agree based on the “essentiality” interpretation. Therefore, we avoided the use of “chimeric” to refer to the virus bearing “foreign”, “heterologous” or “recombinant” sequences. We have substituted this term along the paper.
• The presented results do not justify the claim as stated in the title, since protection against BT has not been shown. Essentially, we agree in that no useful protection from BTV infection was achieved in the sheep model after a single dose vaccination. However, our clinical, virological and immunological data, point towards a vaccine-mediated disease-ameliorating effect. This effect was more notable for the virus expressing NS1Nt in sheep and for both recombinant viruses in the mouse model, at least indicating that triggering (priming) immune responses against the heterologous antigens was possible. Testing experimental vaccine strategies for a first time in a large animal model often generates data that are far from ideal, warranting further improvements and optimization. Nonetheless, we are absolutely convinced that the observations presented in this article may contribute to improve our knowledge of the potential of RVFV as an antigen delivery system. They also indicate that the intrinsic properties of the foreign antigen expressed in the context of a RVFV infection may condition the success of immunization. BTV antigens VP2 and NS1 were selected since they proved relevant for directing protective immune responses against BTV, using more conventional antigen delivery systems. Based on recent data in our lab we have seen much better expression profiles with other potential viral vaccine antigens. This indicates that some BTV antigens, in particular the VP2 protein, can be hard to express in a stable full-length conformation and will need more optimization studies in order to enhance stability and immunogenicity upon infection. In any case, we have followed the suggestion of the reviewer and have changed the title of the article avoiding the term “protection”.
• “Conclusions/Significance” This reviewer has doubts about this prospect based on the presented data and the hurtles to be taken in order to achieve full protection for the added one, here BTV.

We have qualified this remark in the revised version. We just mention here that our data support further developments for improving and optimise, as commented above. Such further developments may include modifications to achieve more stable/sustained VP2 expression levels and improving the challenge model in sheep as well. In any case, we believe that our data represent a promising start to consider recombinant RVFV as an specialized antigen expression platform in areas were RVF vaccination is desirable but difficult to implement. We think the data presented here may open new ideas, give more insights and enhance our understanding to improve the use of RVFV as a potential vector. We expect that further innovations will avoid the limitations found. Our lab is currently working towards this goal.
In addition:
1) Large parts of the world (Australia, Europe, Americas) are RVF-free at the moment. Here, authorities will be very reluctant to permit the use of a RVFV-vector based vaccine for protection against BT or any other disease. Easy serology-based monitoring to demonstrate freedom of RVF could be a problematic after use of such vector vaccine. We agree with the fact that in RVF-free areas preventive RVF vaccination may be preferred using a different type of vaccine with DIVA features. The use of a RVF-vector based vaccine would need to provide methods to easy discrimination of vaccinated animals, not to be confused with RVFV infection. We have novel developments that indicate this should be possible but these findings will be the matter of a new paper.
2) RVF endemic parts of the world will be reluctant too, since the start of an epidemic is harder to notice due to the same reason. Further, this RVFV vector is potentially transmittable to and by insect vectors. Another potential risk is exchange of the fragment with the foreign gene with wild type RVFV, although this will not lead to virulent RVFV. As a consequence, uncontrolled spread of RVFV-vectored vaccine or segments thereof cannot be excluded. In RVF-endemic areas the situation is quite different. Currently, live attenuated vaccines are licensed in Africa. A recombinant vaccine as ours would be also easily traceable. The potential transmissibility of an attenuated vaccine by insect vectors is yet to be demonstrated. Uncontrolled spread, therefore appears at this moment highly speculative. A recent article concluded that “there is no evidence that reassortment between live-attenuated vaccines (LAVs) and wild-type RVFV has occurred during field use, that such a reassortment event if it occurred would have no untoward consequence, and that the Target Product Profiles should be revised (by WHO) to provide a more balanced assessment of the benefits versus the theoretical risks of reassortment.” (see Monath et al Vaccine X. 2020 Apr 9;5:100060. doi: 10.1016/j.jvacx.2020.100060. eCollection 2020 Aug 7). Nonetheless, our lab is working to reduce all these potential drawbacks by generating viruses with lower level of replication in mosquito cells, carrying attenuating mutations in all segments and enhancing polymerase fidelity to reduce the onset of revertants. Using these strategies, we have recently generated recombinant RVFV that are not virulent in A129 mice.
3) Complete protection against BT induced by the presented bivalent vaccine will require boost vaccination(s). The pre-existing immune responses against the viral vector, here attenuated RVFV or a high seroprevalence due to a previous RVFV infection, will reduce the outcome of boost vaccination against BTV. Obviously, the vaccine should be applied to vaccinate against RVFV as well, not only for BTV. Therefore RVF naïve animals would be first choice vaccination targets. Nonetheless, the possibilities of a dual BT-RVFV vectored vaccine are several. It would be possible to boost immune responses in BT vaccinated animals or to prime naïve animals for a secondary BTV vaccine boost. In both cases protection against RVFV would be ensured.
4) In particular for Bluetongue, how to cope with multi-serotype situations in the field with such bivalent RVFV-vectored vaccine for BT? This is a very important question. The NS1 antigen has some intrinsic capabilities for serotype cross-protection as stated in the paper and demonstrated previously, (see Marin-Lopez et al “CD8 T Cell Responses to an Immunodominant Epitope within the Nonstructural Protein NS1 Provide Wide Immunoprotection against Bluetongue Virus in IFNAR(-/-) Mice J.Virol 92, 16 (2018). We envisage that the best BT vaccine would be the one providing multiserotype protection. Our recombinant vaccine encoding BTV NS1 would be useful to enhance multiserotype protection in BT vaccinated sheep.

Reviewer #2: Partial protection of sheep against BTV by rZH548-ΔNSs::VP2BTV-4, or rZH548- ΔNSs::NS1NtBTV-4 was not sufficiently supported by the experiment. We think this may depend on how partial protection is defined. In the absence of a standard threshold we thought that the differences found might be indicative of a partial (or incomplete) protection levels. The finding of lower anti-BTV VP7 titers in vaccinated sheep upon challenge was particularly convincing for us. On the other hand, reduced viremia levels would help to impair viral acquisition by *Culicoides* bites since the efficiency of infection of midges is dose-dependent (i.e the 50% Midge Alimentary Infective Dose (MAID50) was estimated to a blood meal titer around 2×10^5^ TCID50/ml). Therefore by lowering the viremia levels disease spread by insect bites could reduced.

Reviewer #3: Conclusions are definitely supported by the data presented. There is some discussion of how the data can be helpful to advance understanding of the topic. Discussion touches on limitations of the analysis. Public health relevance is not addressed directly.

Reducing the risks of RVF outbreaks in animals has obvious public health consequences in RVF endemic countries. To avoid an extension of the Discussion (as demanded by the reviewers) we just have included the word “human” in the first sentence.

--------------------

**Editorial and Data Presentation Modifications?**


Use this section for editorial suggestions as well as relatively minor modifications of existing data that would enhance clarity. If the only modifications needed are minor and/or editorial, you may wish to recommend “Minor Revision” or “Accept”.

Reviewer #1: Minor comments
• L59-60. Live-attenuated RVF vaccine strain Clone 13 is NOT completely safe, and thus not completely safe as viral vector for vaccine development!! Makoschey et al. Rift Valley Fever Vaccine Virus Clone 13 Is Able to Cross the Ovine Placental Barrier Associated with Foetal Infections, Malformations, and Stillbirths. PLoS Negl Trop Dis. 2016;10(3):e0004550. Clone 13 is not completely safe for pregnant ewes, however note that in those experiments the ewes were overdosed. It would have been interesting to see the effect of a normal dosage inoculation. Nonetheless, by adding more attenuating mutations to the vector we might be able to reduce residual virulence to a maximum level, adding confidence to the use of a recombinant RVFV as vaccine.
• L97-98. Refences of these BTV-4 strains?? BTV-4M is a reassortant (L25-L523)!! Why using this strain as challenge inoculum?? How lethal/virulent is BTV-4M? We have referenced this strain in the revised version and the reason for using BTV4-M was to ensure sheep infection. BTV-4M (BTV serotype 4 strain Morocco (MOR2009/09) is a reassortant strain between BTV-1 (segments 1, 4, 5, 7, 9, 10) and BTV-4 (segments 2, 3, 6, 8) isolated from sheep blood in KC insect cells and not previously passed through mammalian cell lines. This strain maintains virulence in sheep, in contrast to other strains isolated in eggs and grown in mammalian cells such as BTV serotype 4 (SPA2004/02) also used in this work.

• L342-343. This is confusing upon Fig 2C. The arrows indicate very minor bands in lane 2 compared to other nonspecific?) bands and the 25 kDa band, while bands in the right lane are not linked to BTV proteins. We have corrected the figure to add arrows were needed, particularly to indicate the BTV specific protein bands.
• L344-360 can be shortened and Fig 3 can be supp Figure as live-attenuated RVFVs are all similar, while the ancestor or empty live-attenuated RVFV is missing in this figure. This paragraph has been shortened as suggested. We did not generated an empty (ΔNSs) RVFV, since we thought that a better control for our immunization experiments would be a recombinant virus carrying an unrelated antigen in place of the deleted NSs gene (in this case gfp).
• L366-380. Indeed, CPE and IFI are more (most) important as these phenotypic assays show stability of gene expression. Alternatively, full genome sequencing should be performed to demonstrate overall genetic stability. As mentioned by the reviewer, we expected both CPE and detection by immunostaining as important evidences for the stability of the recombinant viruses. We also added a quick confirmatory test by analysing the ratios of genomic detection of fragments by PCR assay. Though full genome sequencing would be ideal (particularly for characterizing mutations that could arise upon cell culture passages) for the purpose of this work we did not considered strictly necessary since high virus yields allowed the use of low passage virus for all the experiments.
• L380. I assume the rescued virus is passed on cell culture and not the (infected/transfected) cells. That is correct. The rescued virus was subjected to a maximum of 4 sequential passages on cell culture. Moreover, the virus encoding VP2 gene it was plaque purified. We have included more details in the corresponding M&M section.
• L383. RVFV or RVF virus!! Corrected as suggested (RVF virus)
• Sup Fig 2 and Fig 4: D.O.: Likely optical density (OD). Corrected as suggested
• L389-399. The characteristics of the ancestor empty live-attenuated rescued RVFV are likely known, and should be used as control to compare with candidate to study the effect of the VP2 insert. As mentioned above we did not generate the empty (ΔNSs) RVFV, since we thought that a better control for our immunization experiments would be a recombinant virus carrying unrelated antigen (in this case gfp).

• L410-428. Unfortunately, the (n)Abs against VP2 seems to be temporary. Lasting protection, if any, is most unlikely after one shot vaccination. We agree with this observation, which is best observed in the kinetics of nAbs induction in sheep, waning after day 8. This warrants further optimization of the VP2 expression to achieve better nAb responses.
• L436-464. (Fig 6.) This experiment is very artificial, however, indicative that something happened. Still, not very important and should be included in supp. Data. We could not perform a direct challenge over the vaccinated mice, since BALB/C mice are not susceptible to BTV-4 infection. To test if the serum was protective we performed passive transfer in BTV-4 susceptible mice (A129). Passive transfer of antibodies is a recognized assay to test the level of humoral immunity present in serum. We believe it adds important information regarding the relevance of the elicited immunity. Thus, we prefer to maintain figure 6.
• L508-510. Indeed, the GFP variant intended to induce a lower humoral response as can be seen in both Figure 8C and for both tested days in Figure 8B. We agree in the observed trend, but since the differences were not statistically significant, we did not include this observation.
• L525-526. The lower mean temperature for the GFP variant is obvious from day 3 to day 6. I don’t know why. Still, the onset of pyrexia is very similar for all three groups. We observed lower mean temperatures for the NS1Nt variant (orange), not for the GFP one (green). A clear increase in temperatures is observed between days 4 and 5 in all groups. This is consistent with detectable virus replication as shown by rtPCR at day 4. Perhaps the differences in temperature could be due to the differences in viral loads (lower for the NS1Nt variant).
• L529-533. This reviewer did not understand this. All three groups reached Ct values corresponding to >3 log pfu?? Yes, this happened between 6 and 8 dpi. However, the control group (GFP) sustained these values also by 11 dpi.
• L536-538. This reviewer agrees with the statement that all data together suggest that vaccinated groups showed a very slight difference compared to the GFP control group. Though differences are small, the data point out towards lower replication levels. Therefore, we describe these observations as the consequence of an incomplete or partially protective effect. This was further confirmed by the lower induction of anti BTV VP7 antibodies after challenge.
• Supp fig 3A: indeed a very very small difference if any.

It appears that within the first days after inoculation the clinical scores were higher in the GFP group, again pointing towards a beneficial effect of the vaccines. The clinical score was performed by blinded veterinary personnel with subjective observational data, therefore the differences observed may not be well matched quantitatively (ie the weight given to each sign was similar). Overall, the group vaccinated with the control virus had clinical signs that were more evident at early times post-challenge. For the sake of clarity, we have modified the graphics of supp. figure 3A (now supp. figure 4a) with the existing data.
• Supp fig 3B: differences are very marginal, if any could be observed! Only the ALT and AST levels were found altered upon BTV challenge. On the other hand, after attenuated rRVFV inoculation we did not expect many differences among groups.
• In addition, BTV4-M did not cause mortality or severe clinical signs as shown for the (control) sheep vaccinated with the GFP variant. BTV4M did not cause mortality or severe clinical signs in sheep at the infectious doses used in this work. However, after infection, control sheep responded with pyrexia, discharge of mucus and breathing problems.

• L557-581. Taken together, it is questionable whether RVFV is a good choice as viral vector vor vector vaccines for other ruminant diseases, see above. Whether or not RVFV become a future vaccine vector may rely in further optimizations that render successful data in terms of efficacy. We are convinced in the potential of the virus not for a general use but as a dual vaccine strategy against RVF and other prevalent ruminant diseases. It is possible that some virus antigens perform better in terms of inducing protective immunity. As stated above we have expressed with the same approach other antigens from other ruminant pathogens. Novel data would help to better characterize the authentic potential of RVFV as a vector.
• L582-620. I agree that these BTV antigens are the most promising. However, previous studies showed a variety of results suggesting that each antigen will not induce complete protection. At least this has been confirmed in this study. Even more, the results are very disappointing. Likely this is caused by truncated (NS1) and degraded (VP2) proteins. Incomplete proteins mostly show a high turnover, and thus are not presented well to the immune system resulting in a poor response as here shown. We can confirm with the experiments showed in this work that the immune system was primed; therefore, antigenic presentation occurred someway. We could detect specific cellular immune responses as well as antibody induction. We think that protection would have been higher if more antigen was presented and that the lack of stability (of VP2 antigen) may have impeded the generation of stronger and/or sustained immune responses. A potential approach for improving the responses would be the stabilization of the VP2 trimers by ensuring the formation of triskelion-like structures compatible with the induction of higher affinity neutralizing antibodies. We also agree with the fact that these antigens are most relevant in the context of a BTV vaccination approach but expressed alone they are not sufficient to ensure robust protective immune responses. As suggested by one of the reviewers, combining both recombinant virus (and hence both recombinant antigens) in a vaccine would help to improve the protection data since both antigens separately do not ensure enough protection. Our data warrant new experiments to confirm if this is the case.
• In general, the discussion section should be shortened. We have shortened (whenever possible) the discussion section as suggested but maintaining the aspects we considered important.

Reviewer #2: "data not shown" is not acceptable in this journal. We have revised this point as suggested. In particular in line 339 (revised manuscript) “not shown” has been removed and now referred to the Methods sections to explain when plaque assays were performed. In line 397 we refer now these data to a new figure (Supp fig 1D), showing the detection of transgenes by indirect immunofluorescence along a number of sequential passages. Other references to “unpublished data” (see lines 171 and 648 in the tracked changes document) have been removed from the text.

Reviewer #3: Minor revision

--------------------

**Summary and General Comments**


Use this section to provide overall comments, discuss strengths/weaknesses of the study, novelty, significance, general execution and scholarship. You may also include additional comments for the author, including concerns about dual publication, research ethics, or publication ethics. If requesting major revision, please articulate the new experiments that are needed.

Reviewer #1: This manuscript describes the rescue of live-attenuated RVFV by reverse genetics containing and expressing of truncated NS1- or VP2 gene of BTV. In addition to in vitro analyses to confirm expression of the foreign genes, immunization trials in mice and sheep were performed and extensively monitored. In addition, vaccinated ruminant target hosts of RVFV and BTV (sheep) were challenged by BTV4 infection. The authors hypothesize protection of target animals against both diseases by vaccination with such bivalent vaccine.
In general, the results are well presented, but the conclusions are more than once “wishful thinking”. In summary, the results are interesting, although disappointing with respect to vaccine development for Bluetongue. Overall, the manuscript must be significantly rewritten with major revisions, and in particular the interpretations of the results must be strongly weakened before it could be reconsidered for publication in PLOS Neglected Tropical Diseases.

We understand that our data would have been less “disappointing” for readers if more/strong protection was achieved. However, our aim was only to test experimentally the performance of this approach. In our view, the results obtained are truly promising by several reasons. Firstly, we could propagate enough a recombinant RVFV (a negative sense RNA virus) to reach high titers while maintaining the expression of the transgenes. In addition, both transgenes were able to induce specific immune responses in both lab animals and natural target species. In the latter case, anti-VP2 neutralizing antibodies were elicited to protective levels reported by other authors. The lack of full protection upon challenge is indicative of a lack of correlation between the in vitro neutralization data and protection, indicating that something else should be induced to reach protective levels. Though our efficacy data in sheep showed subtle differences with the control group, we still believe that these observed differences are real. We are particularly convinced since the levels of anti-VP7 BTV antibodies upon challenge were clearly augmented in the control sheep and this is probably the most sensitive indicator of virus replication. These data open the way for further experiments such as the use of a combined vaccination approach (as suggested by one of the reviewers) or use in prime boost approaches. Nevertheless, to avoid misinterpretations or false expectations we agree to modify the title of the manuscript, avoiding to focus on protection.

Reviewer #2: The manuscript entitled “Chimeric Rift Valley fever viruses encoding bluetongue virus (BTV) antigens can provide immunity and partial protection against a BTV-4 challenge in sheep” by Moreno S et al. describes that the vaccination with the recombinant ZH548 strain (rZH548) of Rift Valley fever virus (RVFV), which encodes Bluetongue virus (BTV) outer capsid protein VP2 in place of RVFV NSs gene in the S-segment (“rZH548-ΔNSs::VP2BTV-4), confers protective immunity in mice challenged with pathogenic RVFV 56/74 strain. Although immunocompetent mice are refractory to BTV infection, passive transfer of sera derived from BALB/c mice vaccinated with rZH548-ΔNSs::VP2BTV-4 and received an additional booster with 1x10^5 PFU of BTV-4 conferred protection of IFNAR-/- A129 mice against BTV serotype 4 (BTV-4). This study also tested the cellular immune responses to another recombinant RVFV (“rZH548- ΔNSs::NS1NtBTV-4), which encodes BTV nonstructural protein NS1 (partial expression of the N-terminus) in place of RVFV NSs gene in the S-segment, via intracellular cytokine staining or ELISPOT assay. Again, the booster with BTV-4 showed a higher level of CD8 T cell stimulation. The protective efficacy of 1x10^7 pfu doses of rZH548-ΔNSs::VP2BTV-4, or rZH548- ΔNSs::NS1NtBTV-4 against the virulent BTV-4 strain was further tested in sheep at 21 days post vaccination. None of those candidate vaccines showed protection against BTV-4 challenge (pyrexia, viremia), whereas authors claimed partial protection of sheep, due to slightly increased Ct in the qPT-PCR for BTV (~ 1 log10 PFU equivalent). The expression of BTV VP7 derived from challenge virus was apparently lower in vaccinated sheep than the control. Supporting Figure 3B indicates that the vaccination with rZH548- ΔNSs::NS1NtBTV-4 apparently prevented the increase of ALT upon BTV-4 challenge. Overall, the study extensively characterized the immunogenicity and efficacy of two candidate chimeric vaccines for RVF and Bluetongue, which will provide useful information for others considering a similar approach using RVFV NSs gene cassette for heterologous antigen expression.

As above, we think that the lack of more satisfactory efficacy data should not condition the presentation of our data, since we believe the results shown could be of interest for PLOSNTD readers.

Reviewer #3: This manuscript covers describes a large body of work and the idea of making a dual vaccine using the reverse genetics RVFV construct is novel. There are a number of places where some bits of information are lacking. See specific comments below:

Introduction
The introduction is well written and just needs correction of a few grammatical errors – these are indicated as comments in the attached pdf. Sorry, but we are not sure to have this attached pdf.

Methods
Under the section entitled ‘plasmid construction’ – it would be a good idea to refer to 1A and 1B in the appropriate places in the text so that the reader knows which part of the figure to look at.
End of line 126 – ‘y’ - typographical error? Both issues have been modified/corrected as suggested

Results
Figure 1 legend – Restriction enzyme site names – these are italicised I the figure legend but not in other parts of the paper – choose one format and keep it consistent. This has been modified as suggested
Line 330 – insert the size in kDa of the NS1Nt band. This has been modified as suggested
Lin 331 – it wasn’t detected at all! Ie was not detected A faint band corresponding to the expected size of VP2 (110kDa) was pointed by the upper arrow. This is why we state that it was difficult to detect.
Lines 330-332 – you mention in the methods that you did immunoprecipitation for MP12 and NS1Nt as well - what did the MP12 and NS1Nt look like in the immunoprecipitation analysis? MP12 was a negative control for expression of BTV antigens We performed an IP assay with anti-BTV antiserum of MP12 and NS1Nt infected cell extracts and it was similar to the observed western blot NS1Nt band. In the amended figure we have added the IP using NS1Nt alone to compare with VP2.
What are the other bands in the BTV4 lane - where is VP7 and NS1? We have now indicated by arrows the expression of BTV specific proteins
Line 340 – you should probably indicate that you used anti-VP2 as well even though this didn’t work. Anti-VP2 antibodies from immune BTV serum or available anti-VP2 mAbs do not work at all with denatured antigens. In the western blot the recombinant virus encoding VP2 was used as a negative control for the expression of NS1Nt.
Fig3B – the figure quality is not great – I had difficulty in seeing the plaque phenotype difference between GFP and VP2/NS1Nt. Corrected as suggested. We have included better resolution images.
Lines 374-375 - I think these are the wrong way around in the text - according to the figure NS1 is normalised to the L segment and VP2 to the S segment. Thank you for the appreciation. Corrected as suggested.
FigS2 legend – you should indicate what the mock inoculum was. Also, what does D O mean? Maybe you mean OD? We use PBS for mock inoculations. We meant OD. Corrected as suggested.
Line 390 – 392 – I was wondering, what about the NS1Nt? Does it have the same effect as VP2 on the N antibody response? We did not detect differences in the anti-N responses as it was shown in Fig S2.
Line 498 – it would have been interesting to include a group having a co-inoculation both vaccine candidates. This is a very interesting suggestion that we should perform in the future, testing also these recombinant virus either as priming or boosting agents. An important issue with large animal models is the limited space and regulations about the number of animals needed for research experiments. Unfortunately, in this case limited room space precluded the use of additional groups.
Line 520 – Fig 8A legend - I think the orange circles are meant to be orange squares according to the figure legend? Thanks very much for the correction. This has been now corrected as suggested.
Fig8A – what does the first black arrow on day 4 indicate? This arrow was inadvertently included and has been removed from the figure.
Line 503-504 - How do you explain the increase of all of the seroneutralisation titres including the GFP control after the BTV4 challenge?. It is expected an increase in the neutralization titers after an exposure to BTV. Perhaps the level of neut. titers in the VP2 group should have been a bit higher than the other groups. We therefore revised the data and re-titered the sera obtained upon challenge. Essentially, we found similar outcomes. The serum from VP2 vaccinated animals had the highest titers and lower increases were found with the gfp control serum.
You only looked at the response to BTV VP2 and RVFV – what about NSNt ie doing ICCS and/or ELISPOT? Why was it not possible to do this? It was not possible since we had no availability of specific tested reagents at that moment. We plan to have soon protocols to characterize the basic sheep cellular immune response involved particularly since NS1-based BTV vaccines appear to be promising strategies to multyserotype vaccination. It is noteworthy to mention that these analysis in sheep usually drive to unclear conclusions given the outbred nature of the experimental sheep used, so all interpretations should be made carefully and on the basis of well-established principles of sheep immunology.
Supp Fig 3B – if you present all of the biochemical parameters in the figure, you should mention all of them in the results section ie what about tp, ggt and alb - you don't mention these. We have included a sentence to mention that the rest of the parameters did not show a significant change among the vaccinated or challenged group.
Discussion
Line 589 – it would have been interesting to look at NS1 antibodies in the sheep challenge trial experiment too. We looked only to anti-VP7 antibodies since we had a specific ELISA-VP7 test working optimally and we believe the increased levels of these antibodies are a much highly sensitive indicator of the level of replication of BTV in the sheep. Probably the NS1 levels would have been found also elevated and could have acted as an indicator of successful challenge.
Line 682-683 – While I know that it is important to determine what each vaccine candidate contributes to immunity and protection why didn’t you have a group of sheep which received a combination of the 2 recombinant vaccines? As above, we did not have additional animal room space available for more sheep groups. But this possibility remains to be done sooner.

Fig S2 figure legend – you should mention what the mock immunogen is in the figure legend. Corrected as suggested

--------------------

PLOS authors have the option to publish the peer review history of their article ([what does this mean?](https://journals.plos.org/plosntds/s/editorial-and-peer-review-process#loc-peer-review-history)). If published, this will include your full peer review and any attached files.


If you choose “no”, your identity will remain anonymous but your review may still be made public.


**Do you want your identity to be public for this peer review?** For information about this choice, including consent withdrawal, please see our [Privacy Policy](https://www.plos.org/privacy-policy).

Reviewer #1: No

Reviewer #2: No

Reviewer #3: Yes: Ann Meyers
