# Supplementary material for: Recombinant Rift Valley fever viruses encoding bluetongue virus (BTV) antigens: Immunity and efficacy studies upon a BTV-4 challenge
Source: PLoS Negl Trop Dis. 2020 Dec 4;14(12):e0008942. doi: 10.1371/journal.pntd.0008942 (PMC7744063; doi:10.1371/journal.pntd.0008942)
Supplement: Attachment — Submitted filename: Reviewer responsers R2.docx [file pntd.0008942.s009.docx]

**Reviewer's Responses to Questions**

Find below our responses (in red) to the reviewers’ comments

**Key Review Criteria Required for Acceptance?**


As you describe the new analyses required for acceptance, please consider the following:


**Methods**


-Are the objectives of the study clearly articulated with a clear testable hypothesis stated?

-Is the study design appropriate to address the stated objectives?

-Is the population clearly described and appropriate for the hypothesis being tested?

-Is the sample size sufficient to ensure adequate power to address the hypothesis being tested?

-Were correct statistical analysis used to support conclusions?

-Are there concerns about ethical or regulatory requirements being met?


Reviewer #1: yes

Reviewer #2: Authors addressed specific points appropriately. This reviewer has no further comments.

Reviewer #3: yes

--------------------

**Results**


-Does the analysis presented match the analysis plan?

-Are the results clearly and completely presented?

-Are the figures (Tables, Images) of sufficient quality for clarity?


Reviewer #1: yes

Reviewer #2: Authors addressed specific points appropriately. This reviewer has no further comments.

Reviewer #3: I still think figure 2c needs clarification - maybe look at the figure legend and the results presented in the text.
We are not quite sure if we understand what is the clarification needed here. We added an indication in the text to refer to the arrowheads in the figure to look for the presence of putative VP2 protein bands (line 354).
--------------------

**Conclusions**


-Are the conclusions supported by the data presented?

-Are the limitations of analysis clearly described?

-Do the authors discuss how these data can be helpful to advance our understanding of the topic under study?

-Is public health relevance addressed?


Reviewer #1: yes

Reviewer #2: Authors addressed specific points appropriately. This reviewer has no further comments.

Reviewer #3: I agree with one of the other reviewer's comments about the limitation of introducing such a vaccine into RVFV-free regions or RVFV endemic regions. The authors should probably include a sentence or two on these limitations.

We have added a short sentence (see lines 83-85) to bring this consideration to the readers’ attention.

--------------------

**Editorial and Data Presentation Modifications?**


Use this section for editorial suggestions as well as relatively minor modifications of existing data that would enhance clarity. If the only modifications needed are minor and/or editorial, you may wish to recommend “Minor Revision” or “Accept”.

Reviewer #1: Accept

Reviewer #2: Authors addressed specific points appropriately. This reviewer has no further comments.

Reviewer #3: Lines 130-132 – it’s still not quite clear what you did here – please clarify. The procedure has been explained in detail in the Habjan paper (2008) cited by reference 19. In a transfection setting, the three rescue plasmids (vL, vM and vS) (v= viral sense) provide DNA templates for the generation of negative sense RNA (genomic RNA or viral RNA) by cellular poymerase I. The two additional p18 plasmids will provide, upon transcription by cellular pol II and translation, the viral N and L proteins for packaging and replication of the viral genomic RNAs. This has been added to the text (lines 135-138) for better understanding.

Lines 313-321 – you need to refer to figure 1a and b separately. Corrected as suggested

Line 320 – which 5 plasmids? A reference to the Methods section has been included for clarification (lines 326-327)

Lines 347-349 – it’s still hard to distinguish between cytoplasm and aggregated bodies – arrows in the figure might help. We have improved the resolution of the figure. It is possible to see large aggregates of NS1Nt (which can also be seen in the supplemental figure 2D). Also, the signals for RVFV and VP2 antisera merge, and this is not seen for NS1Nt, which is detected in more discrete locations in the cytosol.

Line 354 – supp fig 1A or 1B? Both A and B. Corrected.

Line 351 – isn’t the size of BTV VP2 above 80 kDa? How can you say it was barely detectable if you don’t show this the appropriate MW range in fig 2b?. Figure 2b is presented to show only NS1Nt expression as detected by an anti-V5tag antibody. In this western blot VP2 virus was used as a negative control since the tag is not expressed by the VP2 recombinant virus. Therefore the range of mol. weight markers shown was limited to the size of NS1Nt
Lines 367-368 – in the methods, you indicate the anti-serum used was mouse anti-BTV-4 NS1/VP2/VP7 – how can this detect VP5? The appreciation of the reviewer is right. The serum was raised by immunization of mice with recombinant MVA expressing these three antigens but was then boosted with BTV. We have detailed this fact in the Methods section (Lines 183-186).

Lines 368- 370 – I find this confusing? Detection of VP2 by western blot is not possible since most antigenic epitopes are conformational. Therefore, to check the size of the expressed VP2 transgene we had to use metabolic labelling and immunoprecipitation with anti-BTV serum.

Lines 405-406 – in this section – any reason why you didn’t use the rZH548-ΔNSs as a control?
In this way we reduced the number of mice used since we had previous data showing this virus was not virulent in 129 mice. Should the inoculation of the VP2 or NS1Nt rec viruses had induced morbidity and/or mortality we would have included it as a control to show differences in virulence.
--------------------

**Summary and General Comments**


Use this section to provide overall comments, discuss strengths/weaknesses of the study, novelty, significance, general execution and scholarship. You may also include additional comments for the author, including concerns about dual publication, research ethics, or publication ethics. If requesting major revision, please articulate the new experiments that are needed.

Reviewer #1: Review report manuscript no. PNTD-D-20-00574R1

Title: “Recombinant Rift Valley fever viruses encoding bluetongue virus (BTV) antigens: immunity and efficacy studies upon a BTV-4 challenge”.

Authors: Sandra Moreno, Eva Calvo-Pinilla, Stephanie Devignot, Friedemann Weber, Javier Ortego, and Alejandro Brun.

This revised manuscript describing the rescue of live-attenuated RVFV by reverse genetics expressing truncated NS1- or VP2 gene of BTV has been significantly improved.

The authors are speculating about the potential of this RVF vectored vaccine platform for more endemic ruminant diseases and therewith protecting against RVF. This is an attractive approach to appeal vaccination for this zoonotic disease.

In summary, the results are interesting as the vectored vaccine platform is safe but the efficacy requires significant improvement.

The manuscript is now acceptable for publication in PLOS Neglected Tropical Diseases.

Reviewer #2: Revised manuscript clarified the expression of full-length of VP2 mRNA by RT-PCR. Although it is still possible that proposed recombinant RVFV expressing BTV VP2 is a mixture of truncated S-segment and full-length S-segment, authors described the possibility of VP2 truncation in the text, regardless of true mechanism. In terms of protective efficacy, the candidate vaccine does not confer good protection against BTV challenge, which might indicate the insufficient expression of BTV antigens from RVFV NSs cassette. Nevertheless, the study overall carefully described full set of experiment for potential candidate vaccine, which will be sufficient content for the publication at this point.

Reviewer #3: (No Response)

--------------------

PLOS authors have the option to publish the peer review history of their article ([what does this mean?](https://journals.plos.org/plosntds/s/editorial-and-peer-review-process#loc-peer-review-history)). If published, this will include your full peer review and any attached files.


If you choose “no”, your identity will remain anonymous but your review may still be made public.


**Do you want your identity to be public for this peer review?** For information about this choice, including consent withdrawal, please see our [Privacy Policy](https://www.plos.org/privacy-policy).

Reviewer #1: No

Reviewer #2: No

Reviewer #3: No


Figure Files:
While revising your submission, please upload your figure files to the Preflight Analysis and Conversion Engine (PACE) digital diagnostic tool, https://pacev2.apexcovantage.com. PACE helps ensure that figures meet PLOS requirements. To use PACE, you must first register as a user. Then, login and navigate to the UPLOAD tab, where you will find detailed instructions on how to use the tool. If you encounter any issues or have any questions when using PACE, please email us at figures@plos.org.
 
Data Requirements:
Please note that, as a condition of publication, PLOS' data policy requires that you make available all data used to draw the conclusions outlined in your manuscript. Data must be deposited in an appropriate repository, included within the body of the manuscript, or uploaded as supporting information. This includes all numerical values that were used to generate graphs, histograms etc.. For an example see here: http://www.plosbiology.org/article/info%3Adoi%2F10.1371%2Fjournal.pbio.1001908#s5.
 
Reproducibility:
To enhance the reproducibility of your results, PLOS recommends that you deposit laboratory protocols in protocols.io, where a protocol can be assigned its own identifier (DOI) such that it can be cited independently in the future. For instructions see http://journals.plos.org/plosntds/s/submission-guidelines#loc-materials-and-methods

__________________________________________________
In compliance with data protection regulations, you may request that we remove your personal registration details at any time. (Use the following URL: https://www.editorialmanager.com/pntd/login.asp?a=r). Please contact the publication office if you have any questions.
